# Can counter-advertising exposing alcohol sponsorship and harms influence sport spectators’ support for alcohol policies? An experimental study

**DOI:** 10.1186/s12889-023-15250-5

**Published:** 2023-02-27

**Authors:** Maree Scully, Helen Dixon, Emily Brennan, Jeff Niederdeppe, Kerry O’Brien, Simone Pettigrew, Brian Vandenberg, Melanie Wakefield

**Affiliations:** 1 Centre for Behavioural Research in Cancer, Cancer Council Victoria, Melbourne, Victoria Australia; 2 Melbourne School of Psychological Sciences, The University of Melbourne, Parkville, Victoria Australia; 3 Curtin School of Population Health, Faculty of Health Sciences, Curtin University, Bentley, Western Australia Australia; 4 Department of Communication, Cornell University, Ithaca, New York USA; 5 School of Social Sciences, Monash University, Clayton, Victoria Australia; 6 The George Institute of Global Health, Newtown, New South Wales, Australia; 7 Australian Institute of Family Studies, Southbank, Victoria Australia

**Keywords:** Alcohol, Policy support, Sport sponsorship, Industry denormalisation, Intervention study

## Abstract

**Background:**

Exposure to alcohol advertising and sponsorship through elite sport is associated with harmful use of alcohol. Owing to strong financial and cultural ties between alcohol and sport in Australia, policy action to restrict alcohol sport sponsorship is unlikely to occur without strong public support for change. This study tested whether exposure to counter-advertising exposing industry marketing of harmful products—a technique shown to be effective in tobacco control—promotes higher support for policy change and less favourable beliefs about the alcohol industry among sport spectators.

**Methods:**

A sample of 1,075 Australian adults aged 18–49 years who planned to watch an National Rugby League (NRL) State of Origin series game, featuring prominent alcohol sponsorship, was recruited through an online panel and randomly assigned to one of three conditions: control (neutral advertisement); counter-advertisement exposing alcohol harms; counter-advertisement exposing alcohol sponsorship and harms. Participants completed a pre-test questionnaire and viewed their assigned counter-advertisement multiple times in the 5–7 days before the NRL game. Within four days of watching the game, participants completed post-test measures.

**Results:**

Compared to both the control advertisement and the counter-advertisement exposing alcohol harms, participants who viewed the counter-advertisement exposing alcohol sponsorship and harms were significantly more likely to indicate support for each of four policies aimed at restricting sports-related alcohol marketing, including the complete removal of alcohol sponsorship from sport (51% vs. 32% and 37%). They were also significantly less likely to agree with statements such as “alcohol companies should be allowed to sponsor sport since their products are legal” (39% vs. 63% and 60%) and significantly less likely to report liking alcohol companies in general (38% vs. 59% and 54%). There were no significant differences in policy support or industry beliefs between participants who saw the counter-advertisement exposing alcohol harms and those who saw the control advertisement.

**Conclusion:**

Counter-advertising employing messages that expose and critique the intent and impact of pervasive alcohol sponsorship in sport has potential to bolster public support for policies targeting alcohol sport sponsorship, diminish beliefs supportive of alcohol industry marketing strategies and enhance negative views of alcohol companies and their marketing practices.

**Supplementary Information:**

The online version contains supplementary material available at 10.1186/s12889-023-15250-5.

## Background

Alcohol companies invest heavily in sponsorship of elite sport, with 30 of the leading alcoholic beverage brands globally spending a combined $764.5 million on sport sponsorship in 2018 [1]. In Australia, many of the national sporting organisations, competitions, events and teams have sponsorship arrangements with alcohol brands, particularly in popular sports such as Australian rules football (AFL), rugby league (NRL) and cricket, which account for approximately two-thirds of all alcohol sponsorships [2]. Elite sport sponsorship achieves high reach and strong engagement through live spectatorship as well as those watching on television or other screens [3].

While spectator participation levels at sporting events have been impacted by the COVID-19 pandemic, around one in five Australians aged 15 and over was projected to attend at least one sporting event in 2022 [4]. Additionally, over three-quarters of Australians aged 14 years and older watch some form of sport on television [5], with one survey estimating that a total of 60 million hours of sporting content is consumed by Australians at home per week [6]. Sponsorship of sport is an especially powerful promotional tool for alcohol companies as it can transfer positive image attributes from the sport to the brand and/or product, and can potentially neutralise negative associations (e.g., health and social harms of drinking) [7]. In addition, exposure to alcohol advertising and sponsorship messaging in elite sport promotes increased levels of consumption (including among children) [8], which undermines public health efforts to reduce harmful effects of alcohol consumption in the community.

At present, there are very few restrictions on alcohol advertising, and no restriction of alcohol brand sponsorship of sport, under current legislation and regulatory codes in Australia. For example, while the Commercial Television Industry Code of Practice limits the broadcast of advertisements (ads) for alcoholic products on television to mature and adult viewing classification periods, alcohol brand sponsorship and alcohol product advertising during live sporting events and sports programs on weekends and public holidays are notable exemptions to these time-based controls [9]. Further, the Alcohol Beverages Advertising Code (ABAC), an industry self-regulation code, sets standards for the responsible content and placement of alcohol marketing (e.g., print, outdoor, digital, social media, cinema, television, radio, packaging, point of sale materials, alcohol brand extensions to non-alcohol beverage products and marketing collateral) in Australia [10]. These standards include prohibiting alcohol marketing that has strong or evident appeal to minors or is directed at minors through its placement, depicts alcohol misuse, or portrays the consumption or presence of alcohol as contributing to success or achievement. However, the ABAC explicitly does not apply to sponsorship. The inadequacy of industry self-regulation codes in protecting vulnerable populations was highlighted in an international systematic review, including studies conducted in Australia, which found that content violations are common and that youth are exposed disproportionately to alcohol marketing [11]. Exploitation of the live sport loophole is also evident, with an analysis of free-to-air television alcohol advertising in Australia for 2012 documenting that 87% of alcohol ads during the daytime (6am-8:29pm) were placed in sport TV programming [12]. This same study also observed a higher mean number of alcohol ads per hour in sport TV programs during which such advertising was shown than in those non-sport TV programs during which alcohol advertising was aired.

Given the embedded financial and cultural association between alcohol and sport in Australia, government implementation of controls of alcohol sponsorship of sport is unlikely to occur without strong public support, a known driver of advocacy success and policy action [13, 14]. Recent surveys indicate that around half of Australians support alcohol sponsorships being removed from elite sport [15, 16], suggesting there is considerable scope for improvement. One strategy that could be used by public health advocates to increase public support for policy action in this area is the use of counter-advertisements (hereafter referred to as counter-ads) exposing deceptive or predatory industry practices (e.g., targeting marketing to youth) [17]. Counter-ads focusing on the tobacco industry and its conduct have been shown to be effective in shifting beliefs about the tobacco industry and increasing support for tobacco control policy [18, 19]. While some counter-advertising campaigns critiquing the alcohol industry have run in Australia [20, 21], the United States and the United Kingdom [17], no systematic evaluations have been reported to our knowledge. As such, there is insufficient empirical evidence concerning the efficacy of counter-advertising exposing industry marketing practices in mobilising public support for policies restricting alcohol sponsorship of sport.

The present study aimed to address this gap by testing whether spectators who are shown a counter-ad exposing alcohol sponsorship and harms before viewing an alcohol-sponsored sporting event report (i) higher post-event support for policy restricting sports-related alcohol marketing and (ii) less favourable beliefs about the alcohol industry, compared to spectators shown a control ad. We also tested the counter-ad exposing alcohol sponsorship and harms against a traditional alcohol counter-ad (exposing harms associated with alcohol use) to assess the relative effectiveness of each style of counter-ad on spectators’ level of policy support and beliefs about the alcohol industry.

## Method

### Design and participants

The study design was a pre-post, between-subjects experiment comprising three counter-advertising conditions: (A) control (neutral ad); (B) counter-ad exposing alcohol harms; (C) counter-ad exposing alcohol sponsorship and harms. A sample of Australian adults was recruited by Ipsos from their non-probability online panel (and panel partners). Panel members were eligible to participate if they were aged 18–49 years and planned to watch an upcoming 2021 NRL State of Origin game. The NRL State of Origin series is an elite sporting event in Australia that features extensive alcohol marketing [22]. For example, the 2021 series included alcohol sponsor brand logos on both competing teams’ player uniforms (XXXX beer for the Queensland Maroons and Tooheys New lager for the NSW Blues) and large, superimposed sponsor brand logos on the playing field during the television broadcasts (Victoria Bitter (VB) as the official beer sponsor of the State of Origin). General information about the study was provided to panellists (i.e., that it was about drink products) to obtain their informed consent to participate; however, there was no mention of who had commissioned the survey (Cancer Council Victoria) until the debrief at the end of the study.

Following confirmation of their study eligibility, participants completed a pre-test (baseline) questionnaire, viewed a 30-second version of their assigned counter-ad twice and then reported their cognitive and emotional responses to the ad. Participants were randomly allocated to counter-advertising condition using a least-filled quota pre-programmed procedure set up in the backend of the baseline survey by Ipsos. For each condition, gender (male/female) and age (18–34/35–49 years) quotas were applied to achieve a relatively even distribution of participant characteristics in each condition at baseline. An alcohol consumption screening question was also asked of participants at the start of the baseline survey to obtain an approximate 80/20 split of at least monthly drinkers (cf. irregular/non-drinkers). Participants were invited to complete a short exposure task on each of the intervening days between the baseline survey and the game, which was intended to increase their dose of exposure to the assigned ad. Each day, the task rotated between exposing participants to either a 15- or 30-second version of their assigned counter-ad before asking them to answer a single rating question. To test for effects of the counter-ads on support for policies restricting sports-related alcohol marketing and beliefs about the alcohol industry, participants completed a post-test (follow-up) survey within four days of watching the game. Based on previous experimental studies testing audience responses to sport sponsorship [23, 24], counter-advertising [25, 26] and/or anti-industry media content [27], we expected our intervention to produce small effect sizes (i.e., Cohen’s *d* = 0.22–0.35). Thus, to detect group differences of this magnitude with power of 0.80 (p < 0.05), we aimed to achieve a minimum of n = 326 participants per condition in the final sample.

### Counter-advertising intervention

The counter-ad exposing alcohol harms was from a government-developed educational campaign titled *Know When to Say When*, which ranked highly (top 25%) in terms of motivating reduced drinking in a previous message testing study of 83 existing alcohol harm reduction ads [28]. It depicted real-life scenarios highlighting the social harms of excessive alcohol consumption for the drinker as well as how it affects others around them (e.g., spilling drinks on strangers, getting into a physical fight with friends, losing licence for drink driving).

The counter-ad exposing alcohol sponsorship and harms was developed by a creative agency (*Three Wise Men*) following mixed-methods testing of potential concepts with the target audience. The counter-ad depicted scenes of children going to a sporting event or sitting down to watch sport at home and highlighted the routine exposure they receive to alcohol sponsorship in these settings (e.g., via banners/signage around the sports ground, logos on the field, advertising during the telecast). A male voiceover explains that “Our kids are in training. And who’s training them? The alcohol industry. Alcohol sponsorship covers the sports grounds they go to and is promoted during the sport they watch on TV. They’re being trained to think that sport and alcohol go hand-in-hand. But alcohol causes at least seven types of cancer and 2000 cancer deaths every year. If the alcohol giants keep sponsoring sport, the harm will continue to the next generation. Isn’t it time to kick alcohol sponsorship out of sport?” The end-frame included the tagline “Kick alcohol sponsorship out of sport”, and underneath that was the logo for the Cancer Council (a well-known and respected Australian charity).

The neutral ad (control condition) was an existing ad promoting a laptop computer.

### Measures

The set of outcomes that form the basis of this paper are described below. Other domains measured in the baseline and/or follow-up surveys (e.g., brand awareness; sponsorship recall and recognition; image-based similarity; event-sponsor fit; brand attitudes, preferences and purchase intentions; alcohol harm beliefs; alcohol attitudes; next week drinking intentions) are the focus of a separate paper (manuscript under review).

#### Responses to counter-advertisement

Immediately following counter-ad exposure at baseline, participants rated their cognitive (e.g., believability, relevance to them), motivational (i.e., felt motivated to reduce the amount of alcohol I drink) and emotional (e.g., confusion, surprise) responses to their assigned counter-ad using questions adapted from previous studies [25, 28–31]. Responses were recorded on rating scales ranging from 1 = ‘strongly disagree’ to 7 = ‘strongly agree’ (cognitive) or 1 = ‘not at all’ to 7 = ‘extremely’ (motivational and emotional).

#### Policy support

At follow-up, participants indicated their level of support (1 = ‘strongly oppose’ to 7 = ‘strongly support’) for four proposed policies aimed at restricting sports-related alcohol marketing. Responses were collapsed into ‘support’ (5–7) and ‘neutral/oppose’ (1–4) categories.

#### Beliefs about the alcohol industry

At follow-up, participants indicated the extent to which they agreed or disagreed (1 = ‘strongly disagree’ to 7 = ‘strongly agree’) with three positively framed and two negatively framed statements about alcohol companies﻿. Responses were collapsed into ‘agree’ (5–7) or ‘neutral/disagree’ (1–4) categories. Participants also provided a rating of how they feel about alcohol companies in general on a scale ranging from 1 = ‘I don’t like them at all’ to 7 = ‘I like them a lot’, with responses collapsed into ‘like’ (5–7) and ‘neutral/dislike’ (1–4) categories.

#### Baseline characteristics

Participants recorded their gender, age, residential postcode, highest level of educational attainment, parental status and frequency of drinking alcohol over the last 12 months. Socio-economic status (SES) was determined according to the Australian Bureau of Statistic’s Index of Relative Socio-Economic Disadvantage ranking for Australia using participants’ residential postcodes [32].

### Statistical analysis

Data were analysed using Stata/MP V.16.1 (StataCorp, College Station, Texas). One-way analyses of variance with Bonferroni-adjusted post hoc pairwise comparisons were used to test for differences in participants’ cognitive, motivational and emotional responses to the counter-ad (measured at baseline) by condition. Overall differences in participants’ level of support for each policy proposal were assessed using McNemar’s test. Separate logistic regressions were conducted to test for differences by condition in the proportion of participants who were in support of each policy proposal, agreed with each alcohol industry belief statement and reported liking alcohol companies in general (all outcomes measured at follow-up). Where a significant (p < 0.05) omnibus test for condition was found, pairwise differences were assessed with a Bonferroni correction applied for multiple comparisons. All models controlled for days elapsed between surveys, dose of advertising exposure and game number. Sensitivity analyses were conducted using the original, continuous versions of the policy support and industry belief variables (see Supplementary Material 1). As the pattern of results was generally comparable to those found when using the dichotomous versions of these variables, for ease of interpretation, only the latter are presented in text.

## Results

### Sample characteristics

**Fig. 1:**
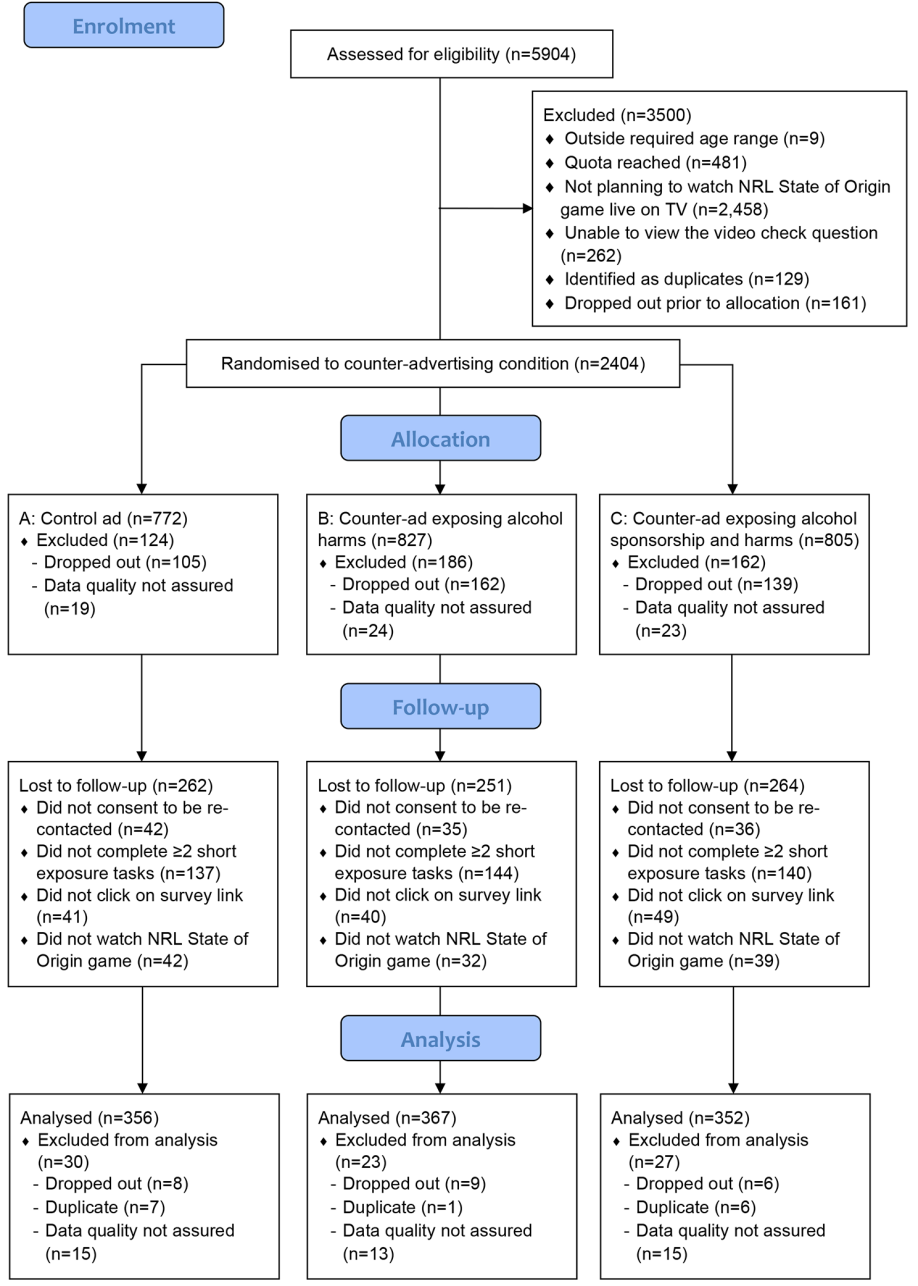
CONSORT flow diagram

The final sample comprised n = 1,075 eligible adults who were recruited and completed data collection (baseline survey, short exposure tasks, follow-up survey) between 2 June and 18 July 2021 (see Fig. 1 for CONSORT diagram). Participants who did not complete at least two of the short exposure tasks (n = 421) or who subsequently reported not watching any of the State of Origin game (n = 113) were excluded a priori from the final sample. On average, participants completed four out of a possible six short exposure tasks (M = 4.3, SD = 1.1) and had an overall dose of advertising exposure across the baseline survey and tasks totalling around two and half minutes (M = 157.6 s, SD = 25.6 s, range = 90–195 s). There was no differential attrition across conditions at follow-up. A summary of the demographic profile of the final sample by condition is provided in Table 1.

**Table 1:** Sample characteristics by counter-advertising condition (n = 1075)

	Counter-advertising condition			
	Total (n = 1075)	Control ad (n = 356)	Counter-adexposingalcohol harms (n = 367)	Counter-adexposingalcohol sponsorship and harms (n = 352)
	%	%	%	%
*Gender*				
Male	52.0	51.7	53.4	50.9
Female	48.0	48.3	46.6	49.1
*Age*				
18–34 years	48.7	49.2	47.7	49.4
35–49 years	51.3	50.8	52.3	50.6
* M (SD)*	34.59 (7.72)	34.24 (7.92)	35.01 (7.67)	34.50 (7.56)
*Highest level of education completed*				
Non-tertiary	45.8	44.7	49.3	43.2
Tertiary	54.2	55.3	50.7	56.8
*SES (area-based)* ^*#*^				
Low SES	23.5	24.7	22.1	23.7
Medium SES	35.9	35.7	36.5	35.4
High SES	40.6	39.6	41.4	40.9
*Parent (any aged child)*				
No	32.5	34.6	32.4	30.4
Yes	67.5	65.4	67.6	69.6
*Frequency of drinking alcohol over last 12 months*				
At least weekly	66.1	66.3	66.5	65.6
At least monthly (but less than weekly)	23.4	22.8	22.6	25.0
Less than monthly / Never	10.4	11.0	10.9	9.4

Notes: Percentages are rounded so may not sum to 100%. All sample characteristics were assessed at baseline^#^ SES was determined according to the Australian Bureau of Statistics' Index of Relative Socio-Economic Disadvantage ranking for Australia using participants' home postcode [32]. Participants who resided in a postcode ranked in the bottom third of the index were categorised as low SES, those in the middle third of the index as medium SES and those in the upper third as high SES. SES information is missing for 2 participants as they provided invalid postcodes

### Responses to counter-advertisement at baseline

As shown in Table 2, both counter-ads were rated significantly higher than the control ad on all cognitive response measures, with the exception that ratings of relevance did not differ significantly for the alcohol harms counter-ad. Participants rated the counter-ad exposing alcohol sponsorship and harms significantly higher than the counter-ad exposing alcohol harms on perceived personal relevance (M = 5.09 vs. M = 3.94, *p* < 0.001), making them stop and think (M = 5.46 vs. M = 5.10, *p* = 0.012) and teaching them something new (M = 5.07 vs. M = 3.99, *p* < 0.001), whereas both counter-ads were rated similarly on ease of understanding, believability and being likely to prompt discussion with others.

**Table 2:** Participants’ cognitive, motivational and emotional responses to counter-advertisement at baseline (n = 1075)

	Counter-advertising condition					
	Control ad (n = 356)		Counter-adexposingalcohol harms (n = 367)		Counter-ad exposing alcohol sponsorship and harms (n = 352)	
	*M*	*SD*	*M*	*SD*	*M*	*SD*
**Cognitive responses**						
Easy to understand	4.33	1.91	6.23^a^	1.08	**6.33** ^**a**^	**1.05**
Believable	4.38	1.70	**6.03** ^**a**^	**1.16**	5.90^a^	1.29
Relevant to me	3.84	1.85	3.94	1.92	**5.09** ^**ab**^	**1.68**
Made me stop and think	3.72	1.79	5.10^a^	1.62	**5.46** ^**ab**^	**1.60**
Would talk to others about	3.33	1.91	4.98^a^	1.65	**5.10** ^**a**^	**1.69**
Taught me something new	3.46	1.88	3.99^a^	1.87	**5.07** ^**ab**^	**1.81**
**Motivational response**						
Reduce the amount of alcohol I consume	2.13	1.78	**4.45** ^**a**^	**1.93**	4.28^a^	2.03
**Emotional responses**						
Surprised	3.03	1.87	3.15	1.79	**4.28** ^**ab**^	**1.70**
Reassured	3.00	1.81	**3.76** ^**a**^	**1.81**	3.48^a^	1.77
Worried	2.05	1.47	3.55^a^	1.82	**4.26** ^**ab**^	**1.75**
Encouraged	3.26	1.92	4.08^a^	1.80	**4.40** ^**a**^	**1.80**
Amused	3.04	1.81	2.77	1.75	**3.01**	**1.85**
Confused	**3.89** ^**bc**^	**2.04**	1.97	1.52	2.20	1.58
Bored	**3.93** ^**bc**^	**1.90**	2.44	1.65	2.62	1.70

oxy_comment_start comment="The footnotes have gotten mixed up in your reordering of the tables. Thus, we have fixed these to match how the tables are currently being shown in the proof. However, please note that our requested changes to the proof in the methods section will mean that the order of the tables in text will revert to how it was in our submitted manuscript. After you have made our requested changes, can you please double-check that the footnotes to each table are correctly matching? If possible, it would be great if we could be sent an updated proof to confirm this for ourselves."Notesoxy_comment_end: Boldfaced figures highlight the counter-advertisement that produced the strongest response among participants. Pairwise differences were assessed using one-way analysis of variance with Bonferroni correction. a Significantly higher than control ad at p < 0.05; b Significantly higher than counter-ad exposing alcohol harms at p < 0.05; c Significantly higher than counter-ad exposing alcohol sponsorship at p < 0.05

Compared to seeing the control ad (M = 2.13), viewing either counter-ad made participants feel more motivated to reduce the amount of alcohol they consume, although these scores were only around the mid-point of the scale (i.e., M = 4.28–4.45). In general, participants’ emotional responses to the counter-ads were moderate (means ranged from 1.97 (‘confused’) to 4.40 (‘encouraged’) on the 7-point scale). However, the counter-ad exposing alcohol sponsorship and harms elicited stronger feelings of surprise (M = 4.28 vs. M = 3.15, *p* < 0.001) and worry (M = 4.26 vs. M = 3.55, *p* < 0.001) than the counter-ad exposing alcohol harms.

### Policy support at follow-up

Across all conditions, participants showed stronger support for a ban on alcohol during sporting broadcasts at times when children watch TV (63%) than a ban on alcohol advertising at sports grounds (47%; McNemar’s χ^2^(1) = 113.88, *p* < 0.001), a policy preventing professional sporting organisations and teams from entering into new sponsorship arrangements with alcohol companies (44%; McNemar’s χ^2^(1) = 134.40, *p* < 0.001) or the complete removal of alcohol sponsorship from sport (40%; McNemar’s χ^2^(1) = 187.72, *p* < 0.001). Compared to both the control ad and the counter-ad exposing alcohol harms, participants who viewed the counter-ad exposing alcohol sponsorship and harms were significantly more likely to indicate support for each of the four proposed policies aimed at restricting sports-related alcohol marketing (see Table 3). Whereas participants who saw the counter-ad exposing alcohol harms recorded similar levels of support for the respective policies to those who saw the control ad.

**Table 3:** Effects of counter-advertising condition on policy support and beliefs about alcohol industry marketing at follow-up (n = 1075)

	Counter-advertising condition			Omnibus test for condition
	Control ad (n = 356)	Counter-ad exposing alcohol harms (n = 367)	Counter-ad exposing alcohol sponsorship and harms (n = 352)	
	%	%	%	
**Policy support (% support)**				
Complete removal of alcohol sponsorship from sport	32.3	36.5	**51.4** ^**ab**^	χ^2^(2) = 29.03, *p* < 0.001
Ban on alcohol advertising at sports grounds	37.9	42.2	**59.9** ^**ab**^	χ^2^(2) = 38.55, *p* < 0.001
Ban on alcohol advertising during sporting broadcasts at times when children watch TV (i.e., before 8:30pm)	54.5	59.9	**74.7** ^**ab**^	χ^2^(2) = 32.61, *p* < 0.001
Policy preventing sporting organisations and teams from entering into new sponsorship arrangements with alcohol companies	36.8	40.6	**53.4** ^**ab**^	χ^2^(2) = 22.22, *p* < 0.001
**Beliefs supportive of alcohol industry marketing (% agree)**				
Alcohol companies make a positive contribution to the community through sport sponsorship	54.8	50.1	**37.8** ^**ab**^	χ^2^(2) = 21.11, *p* < 0.001
Alcohol companies behave in socially responsible ways	42.4	41.4	**28.1** ^**ab**^	χ^2^(2) = 19.98, *p* < 0.001
Alcohol companies should be allowed to sponsor sport since their products are legal	63.2	60.5	**38.9** ^**ab**^	χ^2^(2) = 49.18, *p* < 0.001
**Beliefs opposing alcohol industry marketing (% agree)**				
Alcohol companies are training children to think that sport goes hand-in-hand with alcohol	53.1	56.4	**68.2** ^**ab**^	χ^2^(2) = 19.90, *p* < 0.001
Alcohol companies will stop at nothing to sell their products	58.7	55.6	**66.8** ^**b**^	χ^2^(2) = 9.93, *p* = 0.007
**Overall belief about alcohol companies (% like)**	59.0	54.5	**38.1** ^**ab**^	χ^2^(2) = 33.41, *p* < 0.001

oxy_comment_start comment="As noted above, the footnotes have gotten mixed up in your reordering of the tables. Thus, we have fixed these to match how the tables are currently being shown in the proof. However, please note that our requested changes to the proof in the methods section will mean that the order of the tables in text will revert to how it was in our submitted manuscript. After you have made our requested changes, can you please double-check that the footnotes to each table are correctly matching? If possible, it would be great if we could be sent an updated proof to confirm this for ourselves."Notesoxy_comment_end: Boldfaced figures highlight the counter-advertisement that produced the highest (policy support, beliefs opposing alcohol industry marketing) or lowest (beliefs supportive of alcohol industry marketing, overall belief about alcohol companies) percentage among participants. Logistic regression models controlled for days elapsed between surveys, dose of advertising exposure and game number. Where the omnibus test for counter-advertising condition was significant (p < 0.05), pairwise differences were assessed with a Bonferroni correction applied. a Significant difference compared to control ad at p < 0.05; b Significant difference compared to counter-ad exposing alcohol harms at p < 0.05

### Beliefs about the alcohol industry at follow-up

As shown in Table 3, participants who viewed the counter-ad exposing alcohol sponsorship and harms were significantly less likely to agree with each of the three statements supportive of alcohol industry marketing compared to participants who viewed either the control ad or the counter-ad exposing alcohol harms. They were also significantly more likely to agree with the statement opposing alcohol industry marketing, ‘alcohol companies are training children to think that sport goes hand-in-hand with alcohol’, which was a key message of this counter-ad (68% vs. 53% and 56% respectively). However, agreement that ‘alcohol companies will stop at nothing to sell their products’ was only significantly higher among participants who viewed the counter-ad exposing alcohol sponsorship and harms (67%) in comparison to those who viewed the counter-ad exposing alcohol harms (56%), and not the control ad (59%). The percentage of participants who reported liking alcohol companies in general was significantly lower among those who saw the counter-ad exposing alcohol sponsorship and harms (38%) compared to participants who saw either the control ad (59%) or the counter-ad exposing alcohol harms (54%).

## Discussion

Findings from the present study indicate that counter-advertising exposing alcohol sponsorship and harms has potential to bolster public support for policies to restrict sports-related alcohol marketing, diminish beliefs supportive of alcohol industry marketing and enhance negative views of alcohol companies and their marketing practices. These effects were observed in comparison to both a control ad and a counter-ad highlighting the social harms of excessive alcohol consumption, indicating that it was the specific emphasis on exposing the questionable logic of allowing alcohol companies to promote their product to children during sport that made the counter-ad exposing alcohol sponsorship and harms impactful.

For the four assessed policies to restrict sports-related alcohol marketing, the counter-ad exposing alcohol sponsorship and harms succeeded in boosting support by at least 16% points compared to the control ad and at least 12% points compared to the alcohol harms counter-ad. This is notable given that a previous study gauging public support for 14 alcohol control initiatives across seven countries (Australia, Canada, China, India, New Zealand, United Kingdom, United States) found that support for policies restricting alcohol advertising and sponsorship was typically lower than support for policies related to product labelling and consumer education [15]. Identifying strategies, such as counter-advertising exposing harmful industry marketing practices, that can contribute to redressing this discrepancy is particularly important as robust marketing restrictions are one of the most effective and cost-effective approaches to reducing alcohol-related harm [33–35].

Across the whole sample, the highest level of support among participants was for the policy framed around protecting children (i.e., a ban on alcohol advertising during sporting broadcasts at times when children watch TV), a finding that aligns with past research showing that proposed alcohol control measures that aim to protect young people are better supported by adults than measures targeting the general population [36, 37]. The level of support each policy received from participants in our control condition was quite low in comparison to Australian population surveys. For example, just 32% of control participants were in favour of banning alcohol sponsorship from sport compared to 53% of people aged 14 and over surveyed in the 2019 National Drug Strategy Household Survey [16]. However, this pattern is not altogether surprising as our sample only included younger adults (ages 18–49 years), who have been found to be less supportive of alcohol control policies than older adults [15, 38]. Furthermore, they were a sub-group of younger adult sports spectators, who likely had heavy prior exposure to alcohol sponsorship of sport which could have made them more accepting of this alcohol marketing practice. The fact that exposure to our counter-ad exposing alcohol sponsorship and harms was able to produce such significant increases in policy support among this cohort of young adult sport spectators is encouraging; however, further research is needed to determine if similar effect sizes for this intervention can be replicated in population groups that are already more accepting of government implementing policies to restrict sports-related alcohol marketing.

The counter-ad exposing alcohol sponsorship and harms was effective at dampening specific beliefs supportive of alcohol industry marketing practices, heightening specific beliefs opposing alcohol industry marketing and making participants view alcohol companies less favourably overall. The effect size for this counter-ad detracting from the belief that alcohol companies should be allowed to sponsor sport because their products are legal was particularly large, with percentage point differences of over 20% compared to both the control ad and the counter-ad exposing alcohol harms. These observed shifts in participants’ alcohol industry beliefs in response to seeing counter-advertising exposing and critiquing industry marketing practices are in line with results from a naturalistic experiment where exposure to a movie denormalising the tobacco industry (*The Insider*) promoted more negative perceptions about the industry’s business conduct and less community acceptance of the tobacco industry [27]. They are also consistent with evaluations of the ‘Truth’ campaign in the United States—a youth-focused anti-smoking mass media campaign spotlighting tobacco industry manipulation—that showed associations between campaign exposure and anti-industry beliefs among adolescents [39–41]. Detecting strong effects of our counter-ad exposing alcohol sponsorship and harms on participants’ perceptions of alcohol companies is encouraging, given a demonstrated link between tobacco industry denormalisation beliefs and quitting intentions in adult smokers [42]. Future studies are needed, though, to determine if less favourable beliefs about the alcohol industry are related to reduced drinking intentions.

Assessment of participants’ cognitive and emotional responses to their assigned counter-ad indicated that the counter-ad exposing alcohol sponsorship and harms was perceived as more relevant, thought-provoking and educational (i.e., teaching them something new) than the counter-ad exposing alcohol harms, and also tended to elicit greater surprise and worry. This may reflect participants having had minimal to no prior exposure to public health messages highlighting alcohol industry manipulation tactics, with (to our knowledge) only one previous Australian campaign having used this approach (i.e., Foundation for Alcohol Research and Education’s “Alcohol Truth” social media campaign) [20, 21]. Conversely, messages addressing short-term harms of alcohol have frequently been employed in alcohol harm reduction campaigns [43]; thus, the more familiar theme and tone of the counter-ad exposing alcohol harms could at least partly explain why this counter-ad did not engender as strong a response from participants on these particular measures.

Some study limitations should be acknowledged. First, we only tested a single example of a counter-ad exposing industry marketing practices that in addition to drawing attention to the alcohol industry’s use of sport sponsorship to promote alcohol to children also included mention of a long-term alcohol harm (i.e., cancer) that did not feature in the counter-ad exposing alcohol harms. While this was done to provide context as to why Cancer Council was advocating for the removal of alcohol sponsorship from elite sport, it is not possible to disentangle to what extent the primary (i.e., industry targeting alcohol marketing to youth through sport sponsorship) and secondary (i.e., link between alcohol and cancer) messages of the counter-ad each contributed to the effects we observed. Experimental studies testing multiple examples of counter-advertising exposing alcohol industry practices (including ones without secondary messages) in comparison to other styles of counter-advertising (including ones that focus solely on cancer as a long-term alcohol harm) could provide insight into the features of alcohol counter-ads that most contribute to effectiveness in garnering support for policy change. Second, as this was a naturalistic experiment based around the NRL State of Origin series, our sample was restricted to sport spectators who intended to, and then did watch an event where alcohol sponsorship is typically prominent. An average of 66.29 min (SD = 7.62) of alcohol marketing (including sponsorship) was observed during the two hours of televised coverage of each State of Origin game in 2012 [22], and it is likely that a similar level of exposure occurred during the 2021 series with both teams continuing to feature alcohol sponsor brand logos on their player uniforms. Consequently, participants may have been more likely to be swayed by the counter-ad exposing alcohol sponsorship and harms than non-sport spectators, or those who watch sporting events where alcohol sponsorship is less prominent, given that these participants had the opportunity to see first-hand the widespread promotion of alcohol during the game. Real-world research investigating how the wider population, with varying levels of exposure to alcohol sponsorship of sport, respond to counter-advertising targeting alcohol companies’ use of sport to market their harmful products is an important next step.

Key strengths of the current study include the addition of short exposure tasks that ensured participants received a minimum of four exposures to their assigned counter-ad over a week (i.e., two during the baseline survey and at least two subsequent tasks) to better mimic an actual mass media campaign, and the use of a professionally produced, broadcast-quality counter-ad exposing alcohol sponsorship and harms developed following formative research with the target audience.

## Conclusion

These study findings suggest that counter-advertising exposing industry marketing of harmful products offers a promising avenue for increasing public support for regulatory change in relation to alcohol sponsorship of elite sport and shifting beliefs about the alcohol industry and the acceptability of its marketing practices. Scaling up such counter-advertising to gain wider population exposure would be relatively inexpensive to implement per capita, when compared to the huge social and economic costs of alcohol use in Australia [44]. While the resources available to public health organisations to develop and air counter-advertising campaigns are small relative to the commercial weight of the alcohol industry, this study demonstrates that comparatively brief exposure to counter-advertising with well-designed communications can be impactful. Potential concerns from broadcasters—who benefit financially from alcohol marketing—about running counter-ads that are critical of the alcohol industry could be overcome by disseminating these messages through other digital and social media channels. This type of message delivery strategy has been successfully employed by a 2018-19 Truth anti-e-cigarette campaign (that included anti-industry themes and aired almost exclusively over digital platforms), which achieved high levels of campaign awareness and was associated with higher levels of anti-industry sentiment among the target audience [45]. Thus, such obstacles should not discourage public health organisations from pursuing counter-advertising that exposes and critiques the intent and impact of pervasive alcohol sponsorship in sport given its potential to bolster public support for policy reform in this area.

## Electronic supplementary material

Below is the link to the electronic supplementary material.

Supplementary Material 1

## Data Availability

The data used and analysed in the current study are available from the corresponding author on reasonable request.

## List of abbreviations

NRL: National Rugby League
SES: Socio-economic status
