# Supplementary material for: Can counter-advertising exposing alcohol sponsorship and harms influence sport spectators’ support for alcohol policies? An experimental study
Source: BMC Public Health. 2023 Feb 27;23:396. doi: 10.1186/s12889-023-15250-5 (PMC9969365; doi:10.1186/s12889-023-15250-5)
Supplement: Supplementary file 1 — Supplementary Material 1 [file 12889_2023_15250_MOESM1_ESM.pdf]

Supplementary Material 1: Linear regressions assessing effects of counter-advertising condition on policy support and beliefs about alcohol industry marketing at follow-up (n=1075)

	Counter-advertising condition			
	Control ad (n=356)	Counter-ad exposing alcohol harms (n=367)	Counter-ad exposing alcohol sponsorship and harms (n=352)	Omnibus test for condition
	M (SD)	M (SD)	M (SD)	
**Policy support**				
Complete removal of alcohol sponsorship from sport	3.53 (1.96)	3.80 (1.92)	**4.60 (1.88)**[ab]	F(2, 1068)=28.98, *p*<0.001
Ban on alcohol advertising at sports grounds	3.81 (1.87)	4.23 (1.83)[a]	**4.97 (1.79)**[ab]	F(2, 1068)=35.80, *p*<0.001
Ban on alcohol advertising during sporting broadcasts at times when children watch TV (i.e., before 8:30pm)	4.48 (1.97)	4.81 (1.79)[a]	**5.49 (1.61)**[ab]	F(2, 1068)=29.23, *p*<0.001
Policy preventing sporting organisations and teams from entering into new sponsorship arrangements with alcohol companies	3.80 (1.88)	4.11 (1.75)	**4.70 (1.73)**[ab]	F(2, 1068)=23.28, *p*<0.001
**Beliefs supportive of alcohol industry marketing**				
Alcohol companies make a positive contribution to the community through sport sponsorship	4.61 (1.61)	4.44 (1.54)	**3.86 (1.77)**[ab]	F(2, 1068)=20.44, *p*<0.001
Alcohol companies behave in socially responsible ways	4.24 (1.58)	4.21 (1.54)	**3.66 (1.60)**[ab]	F(2, 1068)=15.54, *p*<0.001
Alcohol companies should be allowed to sponsor sport since their products are legal	4.84 (1.75)	4.75 (1.64)	**3.79 (1.82)**[ab]	F(2, 1068)=39.68, *p*<0.001
**Beliefs opposing alcohol industry marketing**				
Alcohol companies are training children to think that sport goes hand-in-hand with alcohol	4.45 (1.77)	4.58 (1.68)	**5.06 (1.70)**[ab]	F(2, 1068)=13.12, *p*<0.001
Alcohol companies will stop at nothing to sell their products	4.74 (1.63)	4.76 (1.60)	**5.13 (1.63)**[ab]	F(2, 1068)=6.38, *p*=0.002
**Overall belief about alcohol companies**	4.74 (1.37)	4.49 (1.42)	**4.15 (1.37)**[ab]	F(2, 1068)=15.78, *p*<0.001

Notes: Boldfaced figures highlight the counter-advertisement that produced the highest (policy support, beliefs opposing alcohol industry marketing) or lowest (beliefs supportive of alcohol industry marketing, overall belief about alcohol companies) mean response among participants. Linear regression models controlled for days elapsed between surveys, dose of advertising exposure and game number. Where the omnibus test for counter-advertising condition was significant (*p*<0.05), pairwise differences were assessed with a Bonferroni correction applied. [a] Significant difference compared to control ad at *p*<0.05 [b] Significant difference compared to counter-ad exposing alcohol harms at *p*<0.05. All outcomes were measured on 7-point rating scales ranging from 1 = 'strongly oppose'/'strongly disagree'/'I don't like them at all' to 7 = 'strongly support'/'strongly agree'/'I like them a lot'.
